# A Combined Procedure of Intrastromal Corneal Rings Explantation and Penetrating Keratoplasty in a Patient With Keratoconus: A Case Report

**DOI:** 10.3389/fmed.2022.853702

**Published:** 2022-03-11

**Authors:** Raffaele Nuzzi, Flavia Tripoli, Alessandro Rossi, Andrea Ghilardi

**Affiliations:** Department of Ophthalmology, University of Turin, Turin, Italy

**Keywords:** keratoconus (KCN), cross linking, intrastromal corneal ring implantation, penetrating keratoplasty (PKP), cornea

## Abstract

Keratoconus is a non-inflammatory and degenerative corneal ectasia that determinate progressive steepening of paracentral cornea with development of irregular astigmatism and visual function deterioration. According to the stage of the pathology, different methods of correction can be used: rigid contact lenses may be used to alter corneal shape and partially correct astigmatism, corneal collagen cross-linking (CXL) and intrastromal corneal ring segment (ICRS) implantation can reinforce corneal stroma to slow disease progression. Late-stage treatment comprehend anterior lamellar keratoplasty or penetrating keratoplasty. We evaluated a 31-year-old patient who was subjected to bilateral ICRS implantation combined with CXL due to keratoconus. This led, after 9 months, to ring extrusion in his left eye, corneal thinning and microperforation into the aqueous chamber with residual irregular astigmatism of 4.50 D. cyl. 10°. The patient underwent ICRS explantation and PKP during the same surgical session. After 15 months of follow-up, the BCVA was 0.2 LogMAR with a residual astigmatism of 6.3 dpt.

## Introduction

Keratoconus is a non-inflammatory and degenerative corneal ectasia that determinate progressive steepening of paracentral cornea with development of irregular astigmatism and visual function deterioration.

According to the stage of the pathology, different methods of treatment can be used: rigid contact lenses may be used to alter corneal shape and partially correct astigmatism, corneal collagen cross-linking (CXL) and intrastromal corneal ring segment (ICRS) implantation can structurally stabilize corneal ectasia. Late-stage treatment comprehend anterior lamellar or penetrating keratoplasty (1).

Intrastromal corneal ring segment implantation may be considered in patients affected by mild or moderate keratoconus who do not tolerate rigid contact lenses or in case of inadequate astigmatism correction and visual restoration. The two most relevant and frequent postoperative complication of ICRS are functional failure with insufficient correction of ectasia and refractive error, and segment extrusion, with possible damage to corneal epithelium or endothelium (2).

In advanced stages of keratoconus, surgery is often required. Penetrating keratoplasty (PKP) was the first technique to be safely and successfully used to restore visual function. However, graft rejection is a relevant complication that can occur either due to endothelial rejection or progressive dysfunction and failure of endothelial function. More recently, deep anterior lamellar keratoplasty (DALK) was developed and used to treat advanced keratoconus with the great benefit of preserving native endothelial cells and thus reducing rejection risk. Nevertheless, it may lead to serious intraoperative and postoperative complications especially in non-standard cases such as the one presented in this report (3).

## Case Description

We evaluated a 31-year-old patient who at the age of 30 was subjected to CXL and subsequent (after 8 months) ICRS implantation in his left eye due to keratoconus.

He came to our observation 9 months after ICRS implantation complaining about reduced VA in his left eye and great ocular discomfort. The patient did not bring with him any documentation regarding his previous surgery so little to no detail is available. In particular, we could not find any specific evidence regarding the tunnel creation for the ICRS, whether it was done by manual dissection or with femtosecond laser.

Patient’s family history and past medical history was unremarkable. He had no history of atopy but upon request he confirmed that he would frequently rub his eyes, probably due to his work which took place in a dusty environment.

**FIGURE 1:**
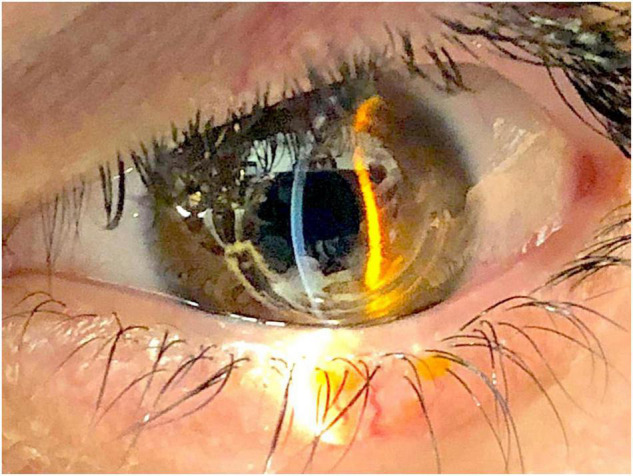
Anterior segment photography showing ring extrusion.

Our examination with the slit lamp evidenced superior ring extrusion, corneal thinning and microperforation into the aqueous chamber (Figure 1). Autorefractometry showed a residual irregular astigmatism of 4.50 dpt cyl. 10°. The uncorrected distance visual acuity of his right eye was 0.0 LogMAR. The UCVA of his left eye was 0.5 LogMAR and the BCVA was 0.4 LogMAR. Due to patient’s intense eye ache and irritation, it was not possible to perform corneal topography or AS-OCT.

We considered that, as of today, there are no data in the literature related to the use of amniotic membranes in corneal extrusion of ICRS: according to our experience acquired over the years, the insertion of the amniotic membrane is to be considered in these cases only a preliminary and transitory intervention. In fact, it only allows for temporary tamponade of the situation and does not allow to avoid a subsequent PKP/DALK intervention (depending on the case) and is not able to re-establish the visual function.

The patient underwent ICRS explantation and PKP in the same surgical session. Firstly, the superior ring was removed, and full thickness corneal perforation was confirmed. After the removal of the second ring, we performed a large diameter PKP to make sure to include the corneal tissue damaged by the segment extrusion. The donor flap diameter was 8.75 mm. We applied two sutures with nylon threads 10.0 and 11.0 to ensure maximal stability of the graft on a host corneal tissue that had received CXL treatment. No complications occurred during the surgical procedure nor afterward in the postoperative follow up.

Twelve months after surgery, nylon suture was removed due to laxity of the treads with no complications.

**FIGURE 2:**
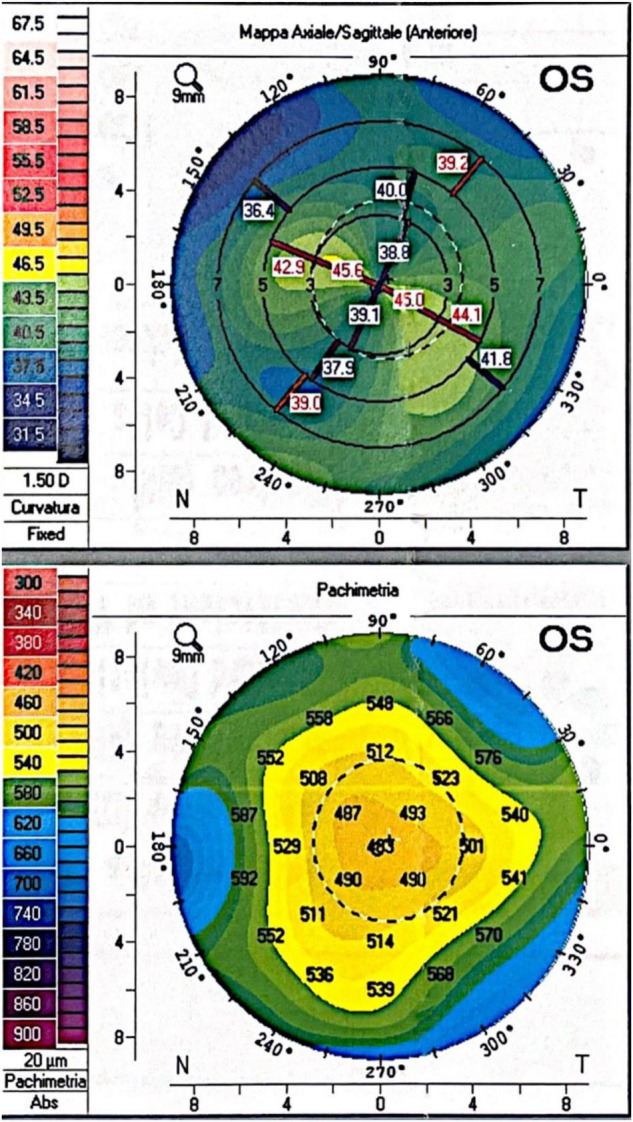
Fifteen months postoperative corneal topography and pachymetry.

After 15 months of follow up, his left eye BCVA was 0.2 LogMAR. His left eye refraction was +3.25 D sph. and −7.00 D. cyl. 60°. The spheroequivalent was −0.25 D. Keratometry and corneal topography were performed with the Pentacam^®^ system. Corneal Km was 41.8 D with an astigmatism of 6.3 D; CCT was 483 μm. Endothelial cell count was 2,117 cells/mm^2^. Topography map and pachymetry are shown in Figure 2.

**FIGURE 3:**
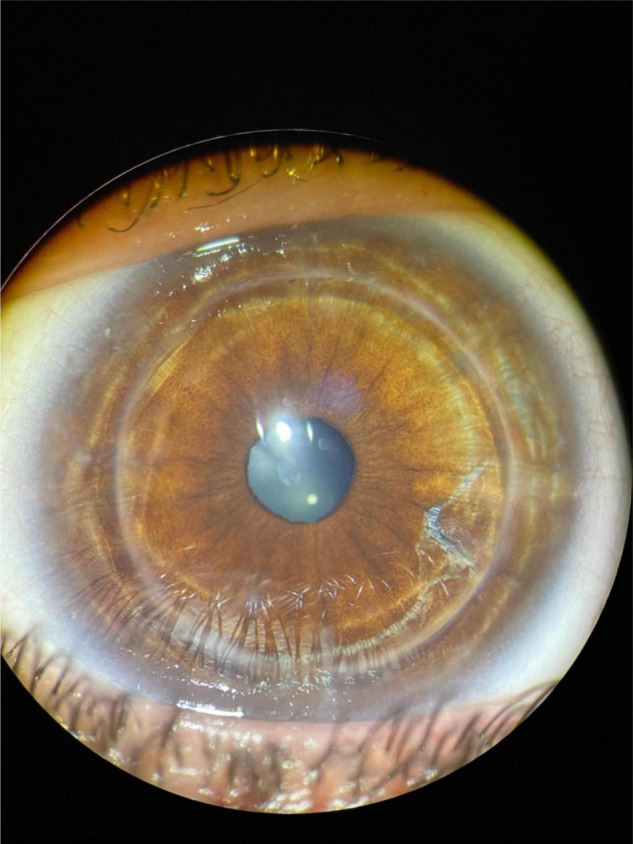
Fifteen months postoperative anterior segment photography.

At the slit lamp examination (Figure 3), the corneal graft was transparent, well centered and adherent. Pupil was centered, round and reactive. Lens was transparent. Fundus examination was unremarkable.

## Discussion

Keratoconus (KCN) is a bilateral non-inflammatory ectasia of the cornea characterized by asymmetric and progressive irregular thinning of the apical and paracentral cornea. With its progression, it may cause high irregular astigmatism and acquired myopia, corneal scarring, acute rupture of Descemet membrane and hydrops with serious visual deterioration (4).

Based on KCN stage, different treatment strategies have been developed.

Apart from rigid contact lenses, frequently used para-surgical techniques include intrastromal corneal ring segments (ICRS) implantation and corneal collagen cross-linking (CXL) (5).

Intrastromal corneal ring segment implantation reinforces corneal stroma, flattens the ectasia, and partially restore corneal symmetry, improving contact lenses correction of astigmatism. ICRS implantation is a reversible procedure, as segments can be explanted in case of complications. CXL is performed to strengthen and compact corneal stroma and prevent or at least to slow the progression of the disease. It has been suggested that combining ICRS with CXL may be beneficial in stabilizing the progression of KCN and to longer preserve visual function. However, such combined technique may be used only if corneal thickness is relatively stable and not rapidly deteriorating. If not planned correctly, it greatly increases the risk of ring extrusion and/or perforation and therefore it should not be recommended or even outlawed as a standard practice. Patients who are candidates to this procedure firstly need to be thoroughly monitored with corneal topography and AS-OCT and a tailored case-by-case approach should be preferred. Alternatively, CXL should be performed after ICRS implantation and only after careful follow-up. In case of disease progression, it may be advisable to skip CXL in favor of PKP or DALK (5). In pediatric age, ICRS are contraindicated, mainly because of easy ring extrusion and important risk of perforation related to growth and development of the eyeball. In such cases, a keratoplasty technique should be preferred (6).

When ICRS implantation is performed, patients should be duly monitored with long-term follow-up due to risk of late complications. Ring extrusion represents one of the most important postoperative complications after ICRS implantation (2) and occurence rate of explantation is is reported in literature to be very low (0–1.4%) (7). The worst scenario is anterior chamber perforation with qualitative or quantitative loss of endothelial cells that may determinate corneal decompensation.

Intrastromal corneal ring segment positioning is extremely important especially in corneas that have also received CXL treatment. If the segment is not properly aligned and the two ends are not positioned at the same depth level in the corneal stroma, we suppose that corneal tissue compaction determined by CXL may be relevant in initiating progressive segment displacement. We argue that first the anteriorization of one end of the ring occurred, followed by progressive anterior extrusion, and subsequently, also due to the intense reactive secondary blepharospasm in a young subject with irregular myopic astigmatism, there was an anomalous inclination of the segment and a perforation of the endothelium in the anterior chamber.

D’Oria et al. (2) performed a multicenter observational case series of ICRS explantation due to different causes. The main reason for explantation was functional failure followed by anatomical failure. In this latter group, spontaneous extrusion was the most common cause, and it was more likely to happen in already advanced cases of keratoconus. Mean extrusion time from implantation is reported to be 25 months.

Late extrusion can also happen, but this apparently does not necessarily prevent good VA restoration. In a case series of patients with late extrusions of ICRS (7, 17, and 20 years postoperatively) it was observed that good BCVA could be preserved after segment removal (8). Furthermore, D’Oria et al. (9) have observed that, even in case of late extrusion, ICRS can be safely removed and that topographic data can return to the preoperative values. Interestingly, patients who had undergone ICRS explantation showed a significant increase in the astigmatic refractive error before extrusion, suggesting that this change might be a predictive and prognostic factor. Unfortunately, we could not retrieve previous topographies of this case, as the patient never brought them to our attention.

Samimi et al. (10) conducted a histopathological investigation of 8 keratoconic human corneas after PKP surgery and ICRS explantation in patients with mediocre visual and refractive outcome or segment extrusion. Their analysis evidenced epithelium hypoplasia, decreased keratocyte number in the site of segment channel and collagen IV synthesis with scar formation. It has therefore been speculated that ICRS implantation may cause keratocyte apoptosis and increased production of metalloproteinase and may accelerate ectasia progression. Kugler et al. (11) have suggested that corneal damage associated with tunnel creation may be a relevant factor in increasing keratocyte apoptosis and it may be associated with a higher number of postoperative complications. Femtosecond laser assisted tunnel creation has been reported to be less traumatic (12); however, there is no definitive consensus whether this method is superior to mechanical tunnel creation and is associated with less complication, extrusion included (2).

While different keratoplasty techniques have been developed throughout the years to selectively treat corneal diseases, the outcome and diffusion of lamellar surgical approaches are still limited by corneal grafting tissue availability, preparation, and quality (13, 14). On the other hand, recent advances in cell engineering techniques may play a relevant role to develop new approaches to increase corneal cell trophism and survival and avoid or delay surgery (15).

In case of anterior extrusion and epithelial perforation, deep anterior lamellar keratoplasty (DALK) or penetrating keratoplasty may be necessary, but only after carefully ruling out anterior chamber perforation and endothelial damage. Furthermore, before attempting a DALK in such complicated cases, corneal topography and AS-OCT should be performed to carefully evaluate corneal thickness. In case of thin and complicated corneas, DALK is contraindicated due to high risk of intraoperative conversion to large diameter PKP with low endothelial sparing, thus enhancing graft rejection. In such cases, PKP should be considered as a reasonable first-line approach.

Penetrating keratoplasty has been the standard technique for surgical treatment of keratoconus. During the postoperative period, graft rejection involving the endothelial layer has been reported to occur with a rate of 20–30% and is the main reason of graft failure and visual deterioration. For this reason, it is also important to evaluate patient’s anamnesis and exclude the possibility of an abnormal and recurrent corneal reactivity (16). DALK has been developed and introduced as an alternative surgical method in the treatment of various corneal conditions, including degenerative diseases such as keratoconus and other stromal dystrophies, and in case of corneal scarring when there is no alteration of the corneal endothelium. The main advantages of DALK over PKP are the reduction of rejection risk involving the corneal endothelium, preservation of endothelium, shorter rehabilitation and reduced postoperative astigmatism. However, DALK diffusion and performance is still limited by technical difficulties in the surgical procedure and steeper learning curve alongside with intraoperative risk of perforation and postoperative complications associated with the graft-host interface (17).

As reported by Kim et al. (17), DALK may be associated with a higher postoperative myopia than the PKP group. This difference may be partially explained by the increased anterior chamber depth (ACD) and higher central corneal refractive power in the DALK group. Henein et al. (18) found that, although DALK has the undeniable advantage of reduced rejection episodes, PKP is still a valid technique in restoring visual function in patients affected by KCN.

High postoperative astigmatism is a common condition after PKP, but it can be safely managed with toric IOL implantation or excimer laser procedures (19).

In conclusion, PKP is still a safe and valid technique in the surgical management of advanced keratoconus and especially in corneas which are particularly thin or complicated, while DALK should be preferred as first line procedure in case of conserved endothelial function and preserved eye anatomy (3).

## Data Availability Statement

The original contributions presented in the study are included in the article/supplementary material, further inquiries can be directed to the corresponding author.

## Ethics Statement

Ethical review and approval was not required for the study of human participants in accordance with the local legislation and institutional requirements. Written informed consent from the patients/participants or patients/participants legal guardian/next of kin was not required to participate in this study in accordance with the national legislation and the institutional requirements. Written informed consent was obtained from the patient for the publication of this case report. All data and images in this article were rigorously anonymized.

## Author Contributions

RN has had full access to the data in this study, drafted the manuscript, and supervised the study. All authors conceived and designed the study, and acquired, analyzed, and interpreted the data.

## Conflict of Interest

The authors declare that the research was conducted in the absence of any commercial or financial relationships that could be construed as a potential conflict of interest.

## Publisher’s Note

All claims expressed in this article are solely those of the authors and do not necessarily represent those of their affiliated organizations, or those of the publisher, the editors and the reviewers. Any product that may be evaluated in this article, or claim that may be made by its manufacturer, is not guaranteed or endorsed by the publisher.
